# Revascularization Strategies for Acute and Chronic Mesenteric Ischemia: A Narrative Review

**DOI:** 10.3390/jcm13051217

**Published:** 2024-02-21

**Authors:** Jacob J. Gries, Takashi Sakamoto, Bing Chen, Hafeez Ul Hassan Virk, Mahboob Alam, Chayakrit Krittanawong

**Affiliations:** 1 Department of Internal Medicine, Geisinger Medical Center, Danville, PA 17822, USA; jgries1@geisinger.edu; 2 Department of Gastroenterological Surgery, Gastroenterological Center, Cancer Institute Hospital of Japanese Foundation for Cancer Research, Tokyo 1130033, Japan; 3 Department of Clinical Epidemiology & Health Economics, School of Public Health, The University of Tokyo, Tokyo 1130033, Japan; 4 Department of Gastroenterology and Hepatology, Geisinger Medical Center, Danville, PA 17822, USA; 5 Harrington Heart & Vascular Institute, Case Western Reserve University, University Hospitals Cleveland Medical Center, Cleveland, OH 44106, USA; 6 Section of Cardiology, Baylor College of Medicine, Houston, TX 77030, USA; 7 Cardiology Division, NYU School of Medicine, NYU Langone Health, New York, NY 10016, USA

**Keywords:** mesenteric ischemia, revascularization

## Abstract

Mesenteric ischemia is a challenging condition characterized by insufficient blood perfusion to the mesentery and, consequently, intestinal tissues that continues to perplex clinicians. Despite its low prevalence, the condition’s variable clinical presentation and elusive radiographic diagnosis can delay life-saving interventions in the acute setting and deteriorate the quality of life of patients when left undiagnosed or misdiagnosed. Purpose: Review and summarize recent diagnostic updates and emergent intervention strategies for acute and chronic mesenteric ischemia. Methods: A narrative review of all relevant studies from January 2022 through September 2023. Results: A total of 11 studies from MEDLINE, supplemented with 44 studies from Google Scholar, were included in the review. Conclusions: Both acute and chronic mesenteric ischemia propose diagnostic and therapeutic challenges for interventionalists. Computed tomographic angiography remains the diagnostic modality of choice for both. Open surgical intervention remains the gold standard for acute mesenteric ischemia, while endovascular techniques are preferred for chronic mesenteric ischemia.

## 1. Introduction

Mesenteric ischemia is a challenging condition characterized by insufficient blood perfusion to the mesentery and intestinal tissues that continues to perplex clinicians. It is classified as “acute” or “chronic” based on the chronicity of hypoperfusion.

Acute mesenteric ischemia encompasses three distinct insult categories that result in diminished blood flow through the mesenteric arteries and ultimately lead to intestinal tissue hypoperfusion [1,2,3,4]. These insults can include arterial embolisms, often resulting from a dislodged thrombus originating in the left atrium, left ventricle, cardiac valves, or proximal aorta [2,3,4]. Additionally, they can stem from acute thrombosis in patients with pre-existing chronic mesenteric ischemia from atherosclerotic disease triggered by abdominal trauma, infection, thrombosed mesenteric aneurysms, and aortic or mesenteric dissections [2,3,4]. Acute mesenteric arterial occlusion accounts for approximately 67–95% of acute mesenteric ischemic cases [3,4]. Other causes of acute mesenteric ischemia include mesenteric venous stasis, which can lead to the formation of venous thrombi [3,4]. This phenomenon can be idiopathic, seen in hypercoagulable states, or secondary to malignancies or prior abdominal surgeries [3,4]. Increased resistance to venous blood flow can result in bowel wall edema, which can promote ischemic events [4]. Nonocclusive mesenteric ischemia is thought to be a consequence of hypoperfusion of the splanchnic vessels and vasoconstriction [3,4]. A recent study found that most cases of acute mesenteric ischemia tend to be caused by occlusions in the superior mesenteric artery, followed by nonocclusive mesenteric ischemia [5].

Despite the low prevalence of acute mesenteric ischemia of only 0.09% to 0.2% of all hospital admissions in the United States [1], the condition’s variable clinical presentation and elusive radiographic diagnosis can delay life-saving interventions in the acute setting and deterioration in the quality of life of outpatients when left undiagnosed or misdiagnosed.

Chronic mesenteric ischemia is distinct from acute mesenteric ischemia, often stemming from peripheral vascular disease where the metabolic demands of the intestines surpass what is supplied [6]. In the fasting state, approximately 20% of cardiac output is directed to the mesenteric arteries [6]. However, postprandial blood flow increases by 100–150% for 3–6 h due to vasodilation of the mesenteric vessels beginning 3 to 5 min after ingestion [6]. Occluded or stenotic vessels in chronic mesenteric ischemia reduce this postprandial hyperemic response, resulting in an oxygen supply and demand mismatch, availability of nutrients, and inadequate removal of waste products [6]. This can lead to symptoms such as pain, malabsorption, and delayed bowel emptying [6].

Although mesenteric artery stenosis is relatively common (affecting up to 10% of the population over the age of 65), chronic mesentery ischemia has a very low incidence, accounting for less than 1 in 1000 hospital admissions for abdominal pain [7]. It affects patients in their 5th through 7th decade and exhibits a strong female-to-male ratio [7]. Patients often present with concomitant manifestations of atherosclerotic disease, including limb ischemia, cerebrovascular disease, and cardiovascular disease, among others. Risk factors that can predispose the mesenteric vessels to atherosclerosis include diabetes, hypertension, tobacco abuse, and dyslipidemia [6,7,8].

Other rare non-atherosclerotic etiologies of chronic mesentery ischemia exist, including fibromuscular dysplasia, median arcuate ligament syndrome, vasculitis, connective tissue disorders, and other uncommon etiologies. Clinical correlation between known diseases and the presentation of abdominal symptoms is essential to establish a diagnosis [9].

A recent study demonstrated that patients with chronic mesenteric ischemia may also have intestinal dysbiosis, which can resolve postoperatively [10]. This study underscores the importance of adequate intestinal perfusion for gut homeostasis and microbiome modulation [10].

The diagnostic approach and management of acute and chronic mesenteric ischemia differs based on the condition’s chronicity, with evolving research reshaping conventional practices. This review will explore common clinical presentations of acute and chronic mesenteric ischemia and explore recent studies regarding their diagnostic protocols and preferred medical and surgical interventions. Furthermore, it proposes unique, comprehensive algorithms to assist with early diagnosis and intervention of acute and chronic mesenteric ischemia. The primary aim of this narrative review is to synthesize recent studies that have challenged traditional diagnoses and interventions to aid quick diagnoses and effective treatment of mesenteric ischemia with hopes of mitigating the high morbidity and mortality rates associated with the condition.

## 2. Methods

**Figure 1:**
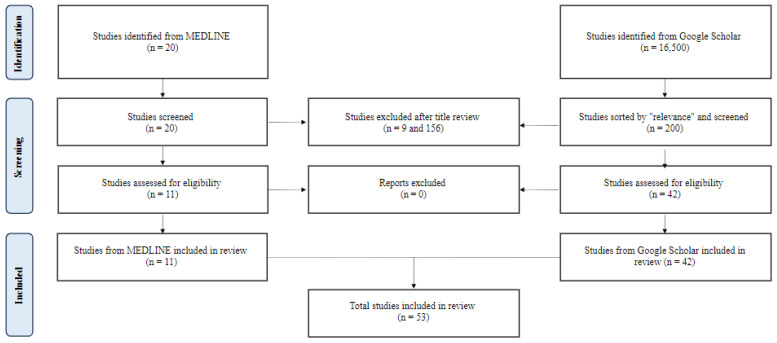
A flow diagram of study selection.

**Figure 2:**
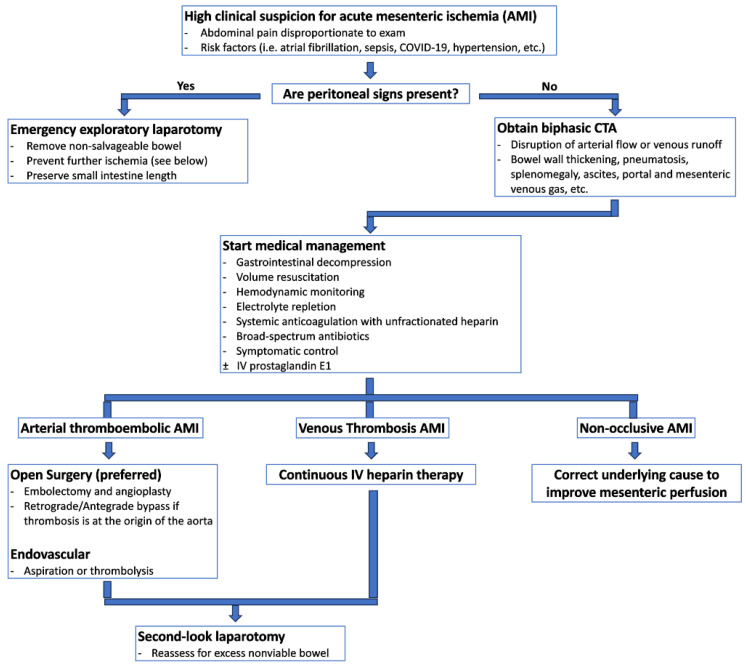
Suggested diagnostic and therapeutic pathway for acute mesenteric ischemia.

To identify relevant studies, a comprehensive search of the Pubmed/MEDLINE database was conducted to retrieve relevant articles from January 2022 to September 2023. A combination of Medical Subject Headings (MeSH) terms and text words related to mesenteric ischemia were used. MeSH terms included “mesenteric ischemia”, “acute mesenteric ischemia”, and “chronic mesenteric ischemia”. Only English-language literature involving human subjects was included, and a range of study types, including prospective cohort studies, experimental studies, population studies, meta-analyses, umbrella reviews, retrospective cohort studies, clinical trials, and observational studies, were eligible for inclusion. Due to the relatively low study yield, a supplemental search of Google Scholar using the same search criteria was performed in September 2023. The results were sorted by “relevance”, and the first 20 pages of results were reviewed for inclusion. After screening and data extraction (Figure 1), the authors conducted a narrative synthesis of the studies, with the extracted data summarized into tables for easy comparison and review. Suggested combined diagnostic and therapeutic intervention pathways for acute and chronic mesenteric ischemia were formulated in Figure 1 and Figure 2 based on the results and outcomes of the included studies.

## 3. Results and Discussion

Two hundred twenty papers from MEDLINE and 200 from Google Scholar were reviewed. Fifty-three met the inclusion criteria (Figure 1). Forty-three studies covered acute mesenteric ischemia, including 22 regarding diagnostic aides, four epidemiologic studies, and 17 on intervention strategies. Eight studies covered chronic mesenteric ischemia, including two on diagnostic aides, five on intervention strategies, and one on both. Two studies covered both acute and chronic mesenteric ischemia, including one on diagnostic aides and one on intervention strategies. The studies and their conclusions are listed in Table 1 and Table 2.

## 4. Discussion

### 4.1. Acute Mesenteric Ischemia

#### 4.1.1. Clinical Presentation

Clinical presentation of acute mesenteric ischemia is classically manifested as pain disproportionate to the physical exam findings [11]. As the condition progresses, abdominal tenderness may extend over the bowel wall, coinciding with the onset of necrosis [11]. The duration and characteristics of pain correlate with underlying pathophysiology, with thromboembolic causes often leading to sudden-onset or progressive pain over days, while nonocclusive mesenteric ischemia tends to progress slowly, is not localized, and varies in severity and consistency [11]. Clinical examinations can differ substantially in nonocclusive mesenteric ischemia cases, with unexplained abdominal distention or gastrointestinal bleeding sometimes being the presenting symptoms, particularly in sedated patients in the intensive care unit, in approximately 25% of cases [12].

Patients with acute mesenteric ischemia often exhibit other signs of critical illness, including septic shock, cardiac issues, and respiratory failure, which may necessitate the use of vasopressor medications [12]. Recent studies have correlated sudden-onset pain or pain requiring morphine with acute mesenteric ischemia [13]. Notably, cases of acute mesenteric ischemia have also been documented in patients with COVID-19, which have been attributed to large vessel thromboembolic events and small-vessel thrombosis related to hypercoagulability and fibrinolysis shutdown and have shown higher in-hospital mortality rate compared to those without COVID-19 [14,15]. 

Identifying risk factors for acute mesenteric ischemia can enhance clinical context and facilitate early diagnosis. Recent research has highlighted several demographic characteristics and comorbidities associated with the condition [16]. It is more prevalent in older patients, particularly those with multiple systemic diseases, including cardiovascular disease, endocrine and metabolic diseases, kidney disorders, digestive disease, respiratory conditions, cerebrovascular disease, vascular disease, and cancer [16]. Hypertension, atherosclerosis, and atrial fibrillation have been identified as predominant comorbidities [5]. Furthermore, critically ill patients with non-obstructive mesenteric ischemia prescribed vasoconstricting agents can increase superior mesenteric artery vasoconstriction, potentially contributing to organ infarcts [17]. Interestingly, the role of atherosclerosis as a risk factor for nonocclusive mesenteric ischemia has been debated in the literature [18].

#### 4.1.2. Diagnosis

Early and accurate diagnosis of acute mesenteric ischemia is critical to obtaining optimal outcomes, as delayed diagnosis is a primary factor for the condition’s mortality rate between 30% and 70% [12,19]. Bowel ischemia should be considered for any critically ill patient experiencing unexplained deterioration [12,19]. Figure 2 provides a potential algorithm to assist with early diagnosis and treatment of acute mesenteric ischemia.

While blood work can support clinical suspicion for acute mesenteric ischemia, abnormal laboratory values or often nonspecific and normal values should not deter urgent advanced imaging in cases with high clinical suspicion. Studies have investigated various biomarkers and blood tests and their correlation to patients’ diagnosis, severity, and prognostication of acute mesenteric ischemia. For instance, a CRP to albumin ratio has been identified as a powerful predictor of in-hospital mortality, surpassing white blood cell count, neutrophil to lymphocyte ratio, and lactate levels [20]. However, other studies have indicated that specific markers like L-lactate, D-dimer, leukocytosis, and neutrophil-to-lymphocyte ratio may not have strong predictive value [21]. Elevated lactate levels should increase the clinical suspicion of mesenteric ischemia but can also be nonspecific. An elevated lactate level on admission has been consistently associated with higher mortality rates in acute mesenteric ischemia cases [22]. Additionally, the diagnostic value of D-dimer has been questioned, with studies suggesting it may serve as an exclusionary test but lacks specificity [23]. Elevated plasma I-FABP has been linked to intestinal necrosis in patients with nonocclusive mesenteric ischemia [24].

The presence of peritoneal signs on physical exam is an indication for emergent/urgent abdominal surgical exploration to assess for bowel perforation, necrosis, and other complications [12]. High clinical suspicion for acute mesenteric ischemia in the absence of peritoneal signs necessitates advanced imaging to support the diagnosis further [12,25]. Plain abdominal radiographs can rule out intra-abdominal free air indicative of bowel perforation but are considered nonspecific for intestinal ischemia [1,25]. Computed tomographic (CT) angiography is the recommended initial imaging for patients with a high clinical suspicion of acute mesenteric ischemia [12,25,26]. The World Society of Emergency Surgery and the American College of Radiology strongly advocate for the prompt use of CT angiography, as studies have shown that every 6 h delay doubles mortality rates [21,26]. Oral contrast should not be used as it can obscure the mesenteric vessels and bowel wall enhancement [12,25].

The standard CT protocol for acute mesenteric ischemia typically involves a biphasic approach, with the first phase consisting of thin-slice angiographic phase acquisition through the entire abdomen using a high contrast injection rate [12,27]. Pre-contrast scans can detect vascular calcification, hyper-attenuating intravascular thrombus, and intramural hemorrhage [12]. The following arterial phase with contrast can reveal arterial filling defects within the arteries or regions of infarction within tissues consistent with arterial occlusive disease, which aids in surgical planning [12,27]. The venous phase may reveal hyper-attenuation within the venous system concerning thrombi [12,27]. Multiplanar reconstructions can help determine the origin of the mesenteric arteries [12]. This biphasic protocol has demonstrated high sensitivity (96–100%) and specificity (94–100%) in diagnosing acute mesenteric ischemia [27]. While some studies have explored the benefits of a triphasic CT protocol with the addition of a non-contrast phase, no significant advantages have been identified beyond the traditional biphasic examination [27]. Dual-energy CT is an emerging tool that enhances intraluminal contrast, which can be particularly useful in cases where contrast timing or injection are issues [26,27]. In nonocclusive mesenteric ischemia, CT angiography may reveal bowel ischemia and free fluid in the presence of patent mesenteric vessels [12]. Mesenteric venous thrombosis may present with findings of bowel wall thickening, pneumatosis, splenomegaly, and ascites, with portal and mesenteric venous gas strongly indicating bowel infarction [21].

Several CT radiographical factors for acute mesenteric ischemia have been identified through recent meta-analyses [28,29]. These include bowel wall thinning, decreased or absent bowel wall enhancement, bowel dilatation, pneumatosis intestinalis, portal mesenteric venous gas, and arterial occlusive acute mesenteric ischemia [28,29]. Bowel wall thickening, portovenous gas, and mesenteric venous gas are highly specific for diagnosing transmural intestinal necrosis [28,29]. Decreased or absent bowel wall enhancement and dilatation can be predictors of transmural intestinal necrosis in venous occlusive acute mesenteric ischemia [28,29].

A prospective cohort study has highlighted the presence of colonic involvement in approximately 28% of acute mesenteric ischemia cases, with the right colon being more commonly affected [30]. Wall thickening is the most common CT finding in such cases [30]. Occlusion of the inferior mesenteric artery is a significant risk factor for colonic involvement [30]. Colonic involvement in CT has been associated with increased morbidity and mortality in cases of acute mesenteric ischemia [30].

While magnetic resonance imaging (MRI) can be considered in diagnosing acute mesenteric ischemia and may offer higher sensitivity, CT is preferred due to its availability, speed, and lower cost [25]. If the diagnosis remains uncertain despite CT or MR angiography, catheter-based arteriography is recommended [12]. Doppler ultrasound has limited utility in diagnosing acute mesenteric ischemia due to gas within the bowel lumen and potential limitations in visualizing the mesenteric vessels in larger patients [26].

#### 4.1.3. Management

##### Medical Management

The medical management of acute mesenteric ischemia involves a comprehensive approach involving multiple modalities. Immediate resuscitation includes gastrointestinal decompression, volume resuscitation, hemodynamic monitoring and support, correction of electrolyte derangements, symptomatic control, anticoagulation, and broad-spectrum antibiotics [6,25]. These measures should be treated as temporizing measures to bridge to emergent definitive surgical intervention. Any delay in surgical intervention can be fatal. 

The inflammatory response following bowel wall infarction can lead to extensive capillary leakage [6,31]. Therefore, adequate crystalloid fluid resuscitation is essential to optimize bowel perfusion while awaiting surgical intervention [6,31]. Loss of the mucosal barrier places patients at risk for bacterial translocation, necessitating early initiation of broad-spectrum antibiotic therapy [6,31]. When an occlusive etiology is known or suspected, full-dose systemic anticoagulation with unfractionated heparin should also be initiated before surgical intervention to stabilize existing clots and prevent further propagation [6]. Nasogastric tube decompression and bowel rest should also be started [6].

The medical management of nonocclusive mesenteric ischemia depends on the underlying cause of bowel hypoperfusion [6,12,32]. In such cases, fluid resuscitation remains crucial for optimizing bowel perfusion, maintaining and enhancing cardiac output, and avoiding vasoconstrictive agents [6,12]. In scenarios with unclear etiology, systemic anticoagulation with unfractionated heparin may be considered [6,12]. Alternative therapies like catheter-directed infusion of vasodilatory antispasmodic agents such as papaverine hydrochloride could also be used [6,32,33]. Continuous intravenous infusion of prostaglandin E1 has also shown promise in improving mortality rates in patients with early signs of nonocclusive mesenteric ischemia [12,34]. Recent studies indicate continuous prostaglandin infusion can reduce lactate concentrations and enhance survival in nonocclusive mesenteric ischemia [35].

Antiplatelet and/or anticoagulation are often required following surgical or endovascular interventions, depending on the type of intervention [6]. A recent study found that immediate postoperative parenteral anticoagulant therapy improves prognosis in patients undergoing intestinal resection [36]. However, due to the elevated bleeding risk in the perioperative period, systemic anticoagulation is often delayed for several days. If stenting is performed, dual antiplatelet therapy is recommended for at least one month with subsequent lifelong antiplatelet monotherapy [6]. If mesenteric venous thrombosis is the etiology of ischemia, long-term anticoagulation is indicated due to an often-concomitant hypercoagulable condition. Patients with comorbidities that typically require systemic anticoagulation, such as atrial fibrillation, should be on lifelong anticoagulation [6]. Mesenteric artery patency surveillance is strongly recommended with duplex ultrasounds at regular intervals, including one month, six months, 12 months, and then annually, with contrast imaging usually reserved for recurrent ischemia [6].

A study involving animal models suggested that single-dose pretreatment with albendazole might ameliorate the inflammatory response and increase the ischemia threshold after the induction of a mesenteric reperfusion injury [37]. The study reported a reduction in proinflammatory cytokines in treated groups [37]. Additional studies are necessary to corroborate the effect of albendazole in humans before implementation in clinical practice. 

##### Surgical versus Endovascular Revascularization

The mainstay of management of the thromboembolic subtypes of acute mesenteric ischemia involves revascularization of viable bowel tissue, typically achieved through surgery or endovascular approaches, with open surgery being the gold standard of definitive management.

**Table 1:** Review of recent studies (2022–2023) regarding acute mesenteric ischemia.

Reference	Study Design	Data Collection	Study Aim	Author Comments
Zeng et al. (2023) [28]	Systematic review with meta-analysis	n = 1031 cases of AMI from 11 studies	Identify CT-based predictive factors of transmural intestinal necrosis in patients with AMI.	More aggressive interventions, i.e., open surgery, should considered if decreased or absent bowel wall enhancement and bowel dilatation are present on CT images.
Yu et al. (2022) [38]	Retrospective observational study	n = 60 cases of non-occlusive AMI	Determine clinical features of critically ill patients with non-occlusive AMI and risk factors for in-hospital mortality.	Non-occlusive AMI should be higher on the differential for critically ill patients with SOFA scores >8 and abdominal pain in an ICU setting.
Otto et al. (2022) [39]	Retrospective observational study	n = 179 cases of AMI	Investigate clinical predictors of postoperative mortality.	Higher rates of intestinal necrosis can be expected with higher lactate levels, therefore necessitating more aggressive interventions, i.e., open surgery.
Piton et al. (2022) [40]	Post-hoc analysis of the NUTRIREA-2 trial	n = 2410 cases of AMI	Study factors independently associated with AMI in critically ill, ventilated patients with shock randomly assigned to receive enteral nutrition or parenteral nutrition.	Resumption of enteral nutrition should be delayed in patients with shock, anemia, and elevated SAPS II score due to the risk of AMI. Parenteral nutrition should be preferred.
Sumbal et al. (2022) [41]	Systematic review with meta-analysis	n = 10,425 cases of AMI from 51 studies	Highlight predictors of mortality in AMI.	All patients should undergo revascularization as soon as possible if operative or endovascular candidates. Strong consideration for more aggressive interventions, i.e., open surgery, when any of these identified risk factors are present.
Bourcier et al. (2022) [24]	Prospective observational study	n = 61 with suspected non-obstructive AMI (33 patients with confirmed AMI, 27 with intestinal necrosis)	Investigate diagnostic features and accuracy of plasma citrulline and I-FABP to diagnose non-occlusive AMI.	I-FABP may be useful in identifying the presence of intestinal necrosis. Higher levels could be used to determine the need for more aggressive interventions, i.e., open surgery. However, more studies are needed to corroborate these findings.
Bagnacci et al. (2022) [42]	Retrospective observational study	n = 84 cases of non-occlusive AMI	Evaluate CT parameters that predict outcomes of patients with non-occlusive AMI.	Combination of IVC and Delta HU of the bowel wall on CT may be useful in prognostication of patients with non-occlusive AMI. However, more studies are needed to corroborate these findings.
Durak et al. (2022) [43]	Retrospective cohort study	n = 248 patients who underwent emergent/elective bowel resection (85 cases of AMI vs. 163 non-AMI cases)	Investigate the significance of immature granulocyte count and delta neutrophil index in the early prediction of mesenteric ischemia.	Elevated immature granulocyte count and delta neutrophil index may be useful in determining the presence of intestinal necrosis, which would necessitate a more aggressive intervention, i.e., open surgery. However, more studies are needed to corroborate these findings.
Konan et al. (2022) [18]	Prospective observational study	n = 165 critically ill patients (59 cases of non-occlusive AMI)	Evaluate whether abdominal atherosclerosis is a risk factor for non-occlusive AMI.	Known atherosclerosis should not deter clinicians from suspecting non-occlusive AMI in critically ill patients due to the small sample size, despite findings suggesting atherosclerosis is not associated with increased risk.
Monroy et al. (2022) [22]	Retrospective cross-sectional study	n = 74 cases of AMI	Determine the relationship between serum lactate admission levels, extent of bowel necrosis, and mortality.	Higher lactate levels should lend themselves to more aggressive interventions, i.e., open surgery, despite no correlation to bowel necrosis extent.
Sinz et al. (2022) [44]	Retrospective observational study	n = 539 patients undergoing imaging for clinically-suspected AMI (216 cases of radiologically-confirmed AMI, 125 cases of surgically-confirmed intestinal necrosis)	Develop novel prognostic tools for the diagnosis of AMI	More studies corroborating the accuracy and reliabillty of the proposed prognostic tools prior to implementation into clinical practice.
Garzelli et al. (2023) [45]	Retrospective observational study	n = 50 cases of reperfusion injury following AMI endovascular revascularization	Assess the prevalence and risk factors of reperfusion injury following endovascular revascularization.	Reperfusion injury is common but does not seem to affect mortality. The risk of reperfusion injury should not deter interventionalists from pursuing endorevascularization techniques.
Ksouri et al. (2022) [30]	Prospective cohort study	n = 114 cases of AMI independently blindly reviewed by two radiologists	Investigate the prevalence, risk factors, and outcomes of colonic involvement in patients with AMI.	Any indication of colonic involvement on CT imaging should prompt either open surgical or endovascular investigation of the inferior mesenteric artery for thromboembolism due to increased morbidity and mortality.
Deshmukh et al. (2022) [8]	Retrospective observational study	n = 1360 individuals with metabolic syndrome	Evaluate the association between metabolic syndrome and AMI.	The absence or presence of metabolic syndrome should not deter a clinician’s suspicion of AMI, despite findings suggesting metabolic syndrome may not be associated.
Nuzzo et al. (2023) [13]	Prospective cross-sectional study	n = 52 patients with AMI n = 85 controls	Identify clinical factors of AMI that should prompt CT evaluation.	Clinicians should consider the timing and severity of abdominal pain in their clinical assessment for AMI due to increaed association between sudden onset pain and AMI.
Kaçer et al. (2023) [20]	Retrospective case-control study	n = 132 patients with AMI	Examine the prognostic significance of C-reactive protein–albumin ratio in cases of AMI.	This value may be helpful in prognostication or determining if more aggressive interventions, i.e., open surgery, are indicated. More studies are needed prior to implementation into clinical practice.
Topolsky et al. (2023) [17]	Retrospective observational study	n = 90 patients with histopathologically proven non-occlusive AMI	Evaluate the influence of vasoconstrictor agents on signs of vasoconstriction and bowel ischemia on MDCT detected in patients with non-occlusive AMI.	Avoid vasoconstricting agents in patients with non-obstructive AMI due to associated worsened bowel ischemia.
Kanugala et al. (2023) [15]	Retrospective cohort study	n = 370 cases of AMI with COVID-19 n = 17,810 cases of AMI only	Assess the outcomes and identify predictors of mortality of hospitalized COVID-19 patients with AMI.	Patients with COVID-19 should be screened for any clinical findings of AMI due to increased prevalence and higher mortality rates.
Uçaner et al. (2023) [46]	Retrospective observational study	n = 48 cases of AMI	Determine the predicting effects of demographic and clinical data, particularly laboratory parameters including lactate, lactate dehydrogenase, and blood urea nitrogen in the preoperative period in AMI.	More aggressive interventions, i.e., open surgery, should considered if elevated preoperative serum lactate, lactate dehydrogenase, or BUN levels are present.
Wu et al. (2023) [16]	Systematic review with meta-analysis	n = 17,103 cases of AMI from 99 studies	Assess the basic demographic characteristics and prevalence of comorbidities in AMI.	AMI should be considered higher on the list of differential diagnoses in patients with significant risk factors even without typical symptoms.
Jaidee et al. (2023) [29]	Retrospective observational study	n = 49 cases of AMI or small bowel obstruction (23 cases of AMI)	Identify overall CT findings of transmural bowel necrosis, and compare CT findings between AMI and small bowel obstruction.	Increased wall thickness should increase clinical suspicion of AMI in right clinical context, when the diagnosis is not clear.
He et al. (2023) [47]	Retrospective observational study	n = 97 cases of AMI	Assess the association of diffuse reduction of spleen density with posteroperative complications.	Low spleen density was associated with higher rates of postoperative complications. More research is needed to corroborate these findings and assess their clinical indications in larger populations.
Reintam Blaser et al. (2023) [48]	Systematic review with meta-analysis	n = 9914 cases of AMI from 75 studies	Estimate the diagnostic accuracy of all potential biomarkers of AMI.	Serum or urine ischemia-modified albumin, interleukin-6, procalcitonin, and intestinal fatty acid-binding protein had moderate associations with AMI. More research is needed to corroborate these findings and their impact on clinical decision making.
Witte et al. (2022) [49]	Retrospective observational study	n = 64 cases of AMI	Determine the association between AMI and impaired quality of life in long-term survivors.	More aggressive management of comorbid conditions may be indicated following AMI.
Tran et al. (2022) [50]	Retrospective observational study	n = 212 total cases of AMI (99 received early revascularization, 113 late)	Identify hospital-based determinants of delayed revascularization.	Any clinical suspicion of AMI warrants a vascular consultation for earlier intervention and improved outcomes.
Tamme et al. (2022) [51]	Systematic review with meta-analysis	Data from 335 studies with 163 included in analysis.	Clarify the incidence of AMI and its different forms.	AMI continues to be a very rare condition, but early recognition and intervention remain vital to positive outcomes.
Kase et al. (2023) [5]	Retrospective population-based review	n = 577 cases of AMI in Estonia	Contribue to populaiton-based studies on AMI.	AMI continues to be a very rare condition, but early recognition and intervention remain vital to positive outcomes.
Martini et al. (2022) [52]	Retrospective cohort study	n = 53 cases of AMI in Germany	Evaluate clinical characteristics, performed surgical procedures, and outcomes of patients with AMI who underwent emergency abdominal surgery.	All patients should undergo revascularization as soon as possible if operative or endovascular candidates.
Rittgerodt et al. (2022) [35]	Prospective observational study	n = 42 cases pf non-occlusive AMI with concomitant shock	Evaluate intra-arterial vasodilators (i.e., prostaglandin) as a viable therapy for non-occlusive AMI.	Intraarterial prostaglandin infusion improved lactate concentrations and overall survival. More studies are needed in larger cohorts to corroborate findings prior to implementation in clinical practice.
Andraska et al. (2022) [53]	Retrospective observational study	n = 148 cases of AMI n = 259 cases of CMI	Define the predictors of postoperative morbidity, mortality, and patency loss for AMI and CMI.	Open surgery reduces the likelihood of bowel resection, and, therefore, should remain the gold standard of AMI revascularization.
Najdawi et al. (2022) [54]	Prospective observational study	n = 58 cases of AMI	Assess the feasibility and outcomes of endovascularization techniques.	Despite acceptable endovascular revascularization outcomes, open surgery should remain the gold standard revascularization approach for AMI.
Brillantino et al. (2022) [55]	Prospective cohort study	n = 85 cases of AMI (47 one-stage treatments vs. 38 two-stage)	Evaluate the efficacy of the damage control approach by two-step surgical procedure.	Serial laparotomies should be considered protocol for both critically ill and non-critically ill patients due to improved clinical outcomes.
Rebelo et al. (2022) [56]	Restrospective cohort study	n = 44 cases of AMI n = 20 cases of CMI	Analyze the outcome of open surgical, endovascular, and hybrid interventions in AMI and CMI.	Rates of in-hospital mortality between endovascular and open surgery appear to be similar; however, open surgery should remain the gold standard of AMI revascularization.
Proaño-Zamudio et al. (2022) [57]	Restrospective cohort study	n = 1520 cases of AMI	Evaluate the effect of delayed abdominal closure on postoperative morbidity and mortality.	Immediate fascial closure should be performed in all open surgeries for AMI.
Badripour et al. (2022) [37]	Animal study	n = 24 rats with induction of AMI	Evaluate outcomes of mesenteric ischemic reperfusion injury following pretreatment with Albendazole.	Single-dose pretreatment with Albendazole ameliorated the inflammatory response and enhanced ischemia thresholds; however, these results need to be corroborated in randomized controlled trials in humans prior to implementation in clinical practice.
Hou et al. (2022) [58]	Systematic review with meta-analysis	n = 2369 cases of AMI from 39 studies	Evaluate outcomes of endovascular and hybrid interventions.	Mortality rates associated with open surgical treatment, endovascular therapy, and retrograde open mesenteric stenting tend to be similar; however, open surgery should remain the gold standard of AMI revascularization.
Shi et al. (2022) [59]	Retrospective cohort study	n = 41 cases of AMI (14 stent thrombectomy plus aspiration versus 27 aspiration alone)	Investigate the outcomes of stent thrombectomy combined with aspiration versus aspiration alone.	If endovascular revascularization approaches are undertaken, then combination therapies, such as stent thrombectomy plus aspiration, should be performed due to higher clearance rates, reduced adjunctive thrombolysis, and shorter hospitalization.
Wang et al. (2022) [60]	Systematic review with meta-analysis	Data from 18 studies were included in analysis.	Evaluated the efficacy and safety of therapies for superior mesenteric venous thrombosis.	Anticoagulation should be the first-line therapy for AMI due to venous thrombosis, but endovascular intervention could be considered if there is a contraindication for systemic anticoagulation.
Bette et al. (2023) [61]	Retrospective observational study	n = 278,121 hospitalization for AMI from 2010 to 2020 in Germany	Analyze long-term trends of hospitalizations, treatment regimen, and in-hospital mortality of in-patients with AMI.	Current management strategies for AMI have shown effectiveness in reducing in-hospital mortality.
Cirillo-Penn et al. (2023) [62]	Retrospective cohort study	n = 34 cases of AMI who underwent retrograde open mesenteric stenting	Analyze long-term outcomes of retrograde open mesenteric stenting.	Retrograde open mesenteric stenting can be considered a feasible and effective open intervention for AMI.
Magnus et al. (2023) [19]	Restrospective cohort study	n = 67 cases of AMI	Study the mortality and delays of management of patients with AMI.	If delayed revascularization is expected, prophylactic systemic anticoagulation should be initiated.
Liu et al. (2023) [36]	Restrospective cohort study	n = 85 cases of AMI (29 without immediate postoperative parenteral anticoagulation vs. 56 with)	Elucidate the benefit of postoperative parenteral anticoagulation in patients with intestinal resection.	Immediate postoperative parenteral anticoagulant therapy was demonstrated to improve prognosis; however, more studies are needed in large cohort to corroborate findings prior to implementation in clinical practice.
Lemma et al. (2023) [63]	Retrospective population-based review	n = 711 cases of AMI	Assess potentially suitable candidates for intestinal transplantation.	Short bowel syndrome should be avoided as salvage operations like transplantation are only feasible in a minute number of cases.
Habib et al. (2023) [64]	Retrospective observational study	n = 42 cases of AMI treated with retrograde open mesenteric stenting	Assess the results of this approach as a suitable alternative to bypass surgery.	Retrograde open mesenteric stenting can be considered a feasible and effective open intervention for AMI. However, lesion proximity to the aortic-superior meseneteric artery junction should be considered.
Kapalla et al. (2023) [65]	Retrospective observational study	n = 74 cases of AMI (61 open surgery cases vs. 13 endovascular plus laparotomy)	Review outcomes in AMI treatment with an open or endovascular approach in association with laparotomy and to evaluate the endovascular-first strategy.	Conventional open and intraoperative endovascular therapy achieved similar results in patients with indications for laparotomy; however, open surgery should remain the gold standard of AMI revascularization.

**Table 2:** Summary of recent studies (2022–2023) regarding chronic mesenteric ischemia.

Reference	Study Design	Data Collection	Study Aim	Author Comments
Safi et al. (2022) [66]	Retrospective observational study	*n* = 50 cases of CTA-verifed CMI	Assess endoscopic duplex ultrasound as initial diagnostic tool for CMI.	More studies are needed in large cohort to corroborate findings prior to implementation in clinical practice, but this technique could be employed when high clinical suspicion for CMI exists despite other equivocal diagnostic results.
Høyer et al. (2022) [67]	Prospective cohort study	*n* = 476 cases of clinically suspected CMI	Explore the involvement of various mesenteric vessels in total splanchnic blood flow and hepatic vein oxygenation.	Assessment of sphlanchic blood flow and hepatic vein oxygenation could be useful in supporting a diagnosis of CMI in clinically questionable cases.
Deshmukh et al. (2022) [8]	Retrospective observational study	*n* = 1360 individuals with metabolic syndrome	Evaluate the association between metabolic syndrome and CMI.	The absence or presence of metabolic syndrome should not deter a clinician’s suspicion of CMI, despite findings suggesting metabolic syndrome may not be associated.
Lehane et al. (2023) [68]	Retrospective observational study	*n* = 744 cases of CMI (209 open repair vs. 535 endovascular)	Investigate whether long-term survival, reintervention, and value differ between open repair or endovascular modalities.	Treatment modality should be determined by anatomic location of stenosis or patient comorbidities; however, endovascular interventions should be preferred.
Andraska et al. (2022) [53]	Retrospective observational study	*n* = 148 cases of AMI *n* = 259 cases of CMI	Define the predictors of postoperative morbidity, mortality, and patency loss for AMI and CMI.	Endovascular interventions demonstrated improved post-operative morbidity but resulted in early symptom recurrence and re-interventions. An endovascular-first approach with close surveillance should be standard in chronic mesenteric ischemia.
Wolk et al. (2022) [69]	Retrospective observational study	*n* = 36 cases of CMI (21 endovascular vs. 15 open repair)	Review outcomes of open treatment and endovascular treatment.	Endovascular revascularization demonstrated comparable perioperative outcomes with higher technical failure, but open intervention can be distinguished by excellent early and late technical success. Shared decision making should be employed, but an endovascular-first approach with close surveillance should be standard.
Ali et al. (2023) [70]	Systematic review with meta-analysis	Data from 335 studies with 163 included in analysis.	Assess the role, long-term patency, and safety of endovascular revascularization in CMI.	Endovascular techniques demonstrated high technical and clinical success rates and patency rates, but restenosis is common. An endovascular-first approach with close surveillance should be standard in chronic mesenteric ischemia.
Patel et al. (2023) [71]	Cost-effectiveness analysis using transition probabilities	*n* = 20,000 cases of CMI randomized to either open or endovascular intervention	Conduct a cost-effectiveness analysis comparing open versus endovascular techniques.	The cost-effectiveness of a procedure should be weighted less compared to efficacy and feasibility when choosing an intervention modality.
Munley et al. (2023) [10]	Prospective cohort study	*n* = 8 cases of CMI	Assess for intestinal dysbiosis and response to revascularization.	Intestinal dysbiosis, especially increased levels of Bacteroidetes and Clostridia, was demonstrated preoperative, resolved perioperatively, and maintained postoperatively. The clinical reproducibility and implication need to be determined with further trials.
Alnahhal et al. (2023) [72]	Retrospective observational study	*n* = 156 cases of CMI treated endovascularly	Investigate the impact of statins on primary patency rates and all-cause mortality following endovascular management.	A high-intensity statin should be initiated in CMI patients treated endovascularly.

Surgical intervention aims to assess and remove any non-salvageable bowel, prevent further bowel infarction with urgent revascularization, and preserve small intestine length [6,73]. This often involves a midline laparotomy, which allows for direct visualization of the bowel to assess blood flow through the mesenteric vessels, observe abnormal color or appearance of the bowel serosa, and monitor peristalsis [6,73]. Additional techniques such as IV fluorescein, Doppler, or intraoperative laser angiography may also be employed to assess perfusion [6]. 

Visceral revascularization procedures, including embolectomy, thrombectomy, endarterectomy, or bypass, are typically performed alongside bowel resection in acute occlusive mesenteric ischemia [19,32,73]. For embolic etiologies, a transverse arteriotomy followed by standard embolectomy in the superior mesenteric artery is performed, while a longitudinal arteriotomy can be performed if incomplete reperfusion or thrombus is suspected [6,32]. Arterial thrombotic cases often require an autologous venous bypass graft that can originate from the infrarenal or supraceliac aorta beyond the thrombus [6,32]. However, a recent study compared retrograde open mesenteric stenting to bypass surgery and found the in-hospital mortality rates and 1-year survival rates to be higher than with bypass [64]. A serial laparotomy is typically performed and recommended within 24 h after the initial surgery if bowel viability is in question [6,32]. Studies have demonstrated that serial laparotomies offer better clinical outcomes even in non-critically ill patients [55].

Short-bowel syndrome from resected nonviable small intestine from acute mesenteric ischemia is a well-documented adverse outcome associated with poor long-term survival. Intestinal transplantation has been considered in this situation. However, a recent study found only a small number of patients are potentially eligible for intestinal transplantation in these cases [63].

Endovascular interventions have gained popularity in the management of acute mesenteric ischemia due to the reports of lower laparotomy requirements, reduced bowel resection, and lower mortality rates. However, no randomized controlled trials exist comparing open versus endovascular approaches. Endovascular treatment options include percutaneous transluminal angioplasty and stenting, aspiration embolectomy, catheter-directed thrombolysis, or a combination of approaches [6]. Some studies have indicated that combining stent thrombectomy with aspiration versus either technique alone can lead to a higher clearance rate, reduced need for adjunctive thrombolysis, and shorter hospital stays [59]. Retrospective analyses of patients undergoing endovascular recanalization have shown excellent technical success rates and low postprocedural complication rates [54]. These interventions have also demonstrated promising survival rates at three months, one year, and 3 years [54]. A significant drawback of endovascular interventions is that the bowel cannot be visually inspected, and the necrotic bowel cannot be resected. Subsequent open surgical intervention may be required after an endovascular approach. 

Recent nationwide studies in Germany have highlighted trends in treatment approaches for acute mesenteric ischemia. In most patients who undergo mechanical interventions, visceral surgery is preferred over endovascular interventions by a large margin [61]. However, visceral surgery has been preferred, especially in patients with high risks of complications, such as bowel ischemia, perforation, or peritonitis, and has therefore been associated with higher mortality rates [61]. Comparative studies between open and endovascular approaches have shown similar outcomes in patients with indications for laparotomy [53,65]. Logistically, open approaches have resulted in quicker time to intervention [53]. While some small cohort studies have reported better outcomes for endovascular interventions [53,56,58,74], larger database analyses have consistently shown that these interventions are associated with improved postoperative morbidity and mortality, lower rates of in-hospital mortality, lower rates of bowel resection, decreased need for parenteral nutrition, shorter hospital stays, and lower cost compared to open surgery [53,74,75,76].

### 4.2. Chronic Mesenteric Ischemia

#### 4.2.1. Clinical Presentation

Chronic mesenteric ischemia, often called “intestinal angina”, is characterized by relapsing and remitting postprandial pain due to hypoperfusion of the small intestine in patients with multivessel mesenteric artery stenosis or occlusion [7]. Additional nonspecific symptoms include weight loss, food aversion, nausea, vomiting, early satiety, and gastrointestinal disturbances like diarrhea or constipation [7]. A history of other symptoms related to diffuse atherosclerotic disease, such as cardiac angina, cerebrovascular accidents/transient ischemic attacks, or lower extremity claudication, may be present [7]. Physical exams may be vague with diffuse mild abdominal tenderness without rebound or guarding but are often normal for most patients [7]. Abdominal bruits may be auscultated in approximately 50% of the patients but should not be solely relied on for diagnosis [7].

#### 4.2.2. Diagnosis

**Figure 3:**
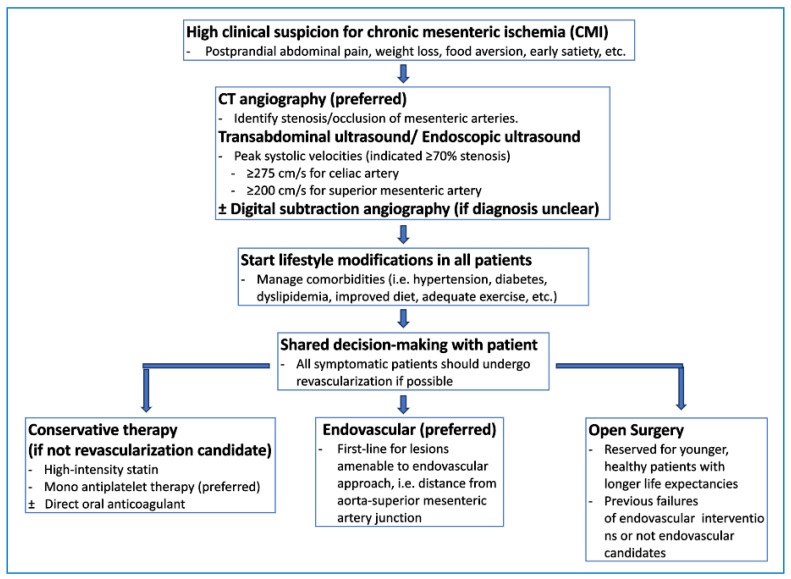
Suggested diagnostic and therapeutic pathway for chronic mesenteric ischemia.

Diagnosing chronic mesenteric ischemia can be challenging due to other more common gastrointestinal diseases that can mimic its symptoms [7]. These conditions include peptic ulcers, cholecystitis, malignancy, and gastroparesis [7]. Vascular imaging is essential to diagnose chronic mesenteric ischemia and to identify stenosis or occlusions of the major mesenteric vessels [7]. Figure 3 provides a potential algorithm to assist with diagnosing and treating chronic mesenteric ischemia.

CT angiography is the preferred initial noninvasive modality, as it can detect stenotic or occluded vessels and rule out alternative pathologies simultaneously [7]. It has a high sensitivity of 100% and a specificity of 95% [77]. Typical CT findings of chronic mesenteric ischemia include stenosis of the mesenteric vessels, thickening of the bowel wall, peritoneal free fluid, and extensive collateral vascularization [78].

Duplex ultrasonography is another reasonable alternative for diagnosis [7]. Peak systolic velocities ≥ 275 cm/s for the superior mesenteric artery and ≥200 cm/s for the celiac artery indicate greater than 70% stenosis and are necessary for diagnosis [7]. The sensitivity of duplex ultrasonography in chronic mesenteric ischemia is 72–100% with a 77–90% specificity [77]. Endoscopic duplex ultrasound has been found to have an even higher sensitivity than transabdominal duplex ultrasound but is not often used clinically and is not considered standard of care [66]. 

Arterial digital subtracted angiography can be used to confirm the diagnosis if it is unclear and therapeutic interventions are planned [7]. This allows for selective catheterization and pressure measurements across stenotic vessels, helping to quantify the degree of hemodynamic significance of these lesions [78].

MR angiography has also been considered an alternative to CT angiography for identifying stenotic or occluded mesenteric vessels, but direct comparisons between the two methods are limited. However, CT angiography is generally shown to be superior to contrast-enhanced MR angiography with regard to the renal arteries [79].

A recent retrospective study developed and tested a novel scoring model to estimate the severity grade of mesenteric artery stenosis [80]. It is a weighted six-point score named the “CSI-score”, with “C” for celiac artery, “S” for superior mesenteric artery, and “I” for inferior mesenteric artery, and based on the number of affected vessels and the extent and grade of stenosis or occlusion of the involved visceral arteries [80]. The study found higher-scoring patients to show statistically higher rates of coronary artery disease, chronic renal insufficiency, and peripheral artery disease [80]. The score stratification showed that many high-score participants underwent endovascular, open surgical, or open conversion after endovascular treatment for chronic mesenteric ischemia [80]. Patients with higher scores also demonstrated a significantly higher mortality rate [80]. The CSI score has shown an excellent ability to predict the degree of mesenteric artery stenosis and a strong association with the need for treatment, open surgery, and mortality [80]. Scoring tools such as these, in addition to imaging findings, patient surgical candidacy, and interventionalist preferences or skills, can aid in determining optimal and individualized therapeutic intervention strategies.

#### 4.2.3. Management

Interventions for patients with chronic mesenteric ischemia aim to relieve symptoms, improve malnutrition, and prevent bowel infarction [77]. Lifestyle modifications are essential and include encouraging patients to manage risk factors such as blood sugar, tobacco cessation, blood pressure control, improved diet, and adequate physical activity [77]. Conservative management often involves antiplatelet therapy and high-intensity statins, but all surgical candidates should undergo revascularization [77]. A meta-analysis comparing dual antiplatelet versus mono antiplatelet therapy after endovascular arterial revascularization found no significant advantage in restenosis or stent thrombosis but showed an increased risk of bleeding with dual antiplatelet therapy [81]. For select patients, direct oral anticoagulants like rivaroxaban may be considered [82]. A study shared that low-dose rivaroxaban with aspirin reduced major adverse cardiovascular and limb events compared with aspirin alone without significantly increasing major bleeding [82].

The Society for Vascular Surgery recommends revascularization for symptomatic patients with chronic mesenteric ischemia, aiming to reverse the presenting symptoms and improve overall quality of life [83]. The primary target for revascularization should be the superior mesenteric artery, with the celiac and inferior mesenteric arteries considered secondary targets [83]. Asymptomatic patients with severe mesenteric artery stenosis should have shared decision-making discussions about the need for revascularization [83]. Endovascular revascularization is strongly recommended as the initial treatment in patients with amenable lesions, with open surgical revascularization reserved for patients with lesions not amenable to an endovascular intervention approach [83].

Patients with untreated symptomatic chronic mesenteric ischemia face a 5-year mortality rate upwards of 100% [77]. Surgical revascularization techniques were initially preferred; however, endovascular approaches have lower rates of in-hospital cardiac and cerebrovascular events, shorter hospital stays, lower in-hospital complications, and better cost-effectiveness [70,71,77]. They have become the preferred treatment modality for lesions amenable to endovascular revascularization [83]. Flush aortic occlusions, small caliber vessels, extensive calcification, tandem lesions, and the degree of stenosis can complicate endovascularization [83].

The skill and preference of the interventionalist usually determine the endovascular technique chosen to restore patency to the stenotic blood vessel [83]. The Journal of Vascular Surgery reports that balloon-expandable covered stents have replaced more traditional balloon angioplasty alone due to improved long-term patency [83]. Post-revascularization, most patients maintain symptom relief for 5–10 years, but restenosis may occur in up to 20–40% of patients and require reintervention [70,77]. High-intensity statins following endovascular intervention have been associated with improved primary patency [72]. Serial duplex ultrasounds are recommended at 3–6-month intervals to monitor for restenosis or symptom recurrence [77]. 

If the vessel is not amenable to endovascularization, then open surgical interventions can be considered [83]. Potential approaches include antegrade bypass from the supraceliac aorta, retrograde bypass from the common iliac artery (or infrarenal aorta), aortic endarterectomy, and open retrograde mesenteric stenting [83]. Limited studies compare the methods directly, but individual studies have demonstrated their significant individual technical and clinical success rates [83]. Several studies have assessed the outcomes of different interventions for chronic mesenteric ischemia. Patient comorbidities often play a significant role in long-term mortality. Factors like congestive heart failure, malignancy, age, and chronic pulmonary diseases have been linked to different mortality rates independent of treatment modality [68]. However, comparative studies have suggested that endovascular revascularization can have similar perioperative outcomes to traditional open surgical interventions but demonstrate higher rates of late technical failure with symptom recurrence and need for reintervention [35,70]. Despite excellent early and late technical success rates associated with open surgery techniques, they have been associated with higher rates of postoperative major adverse events and 30-day mortality [69].

## 5. Conclusions and Future Directions

Both acute and chronic mesenteric ischemia continue to pose a significant challenge to diagnosticians and interventionalists due to the often nonspecific presentations that delay diagnosis and lead to higher mortality rates. Early diagnosis and revascularization are vital to optimal outcomes, improved survivability, and quality of life. A strong understanding of the pathophysiology, diagnostic techniques, and treatment options is crucial for healthcare practitioners to identify the conditions early and optimize outcomes with timely interventions.

Biphasic CT angiographic protocols are essential for the diagnosis of both conditions. Newer studies are continuing to refine diagnostic findings for both conditions. Identifying early signs of transmural intestinal necrosis in acute mesenteric ischemia, such as what has already been recently correlated with bowel wall thinning, decreased or absent bowel wall enhancement, bowel dilatation, pneumatosis intestinalis, and the presence of portal mesenteric venous gas, can aide interventionalists in determining the optimal treatment modality for the patient. Further investigations into whether endoscopic ultrasound is a viable diagnostic tool for chronic mesenteric ischemia could also be performed, as it may be a second-like modality if transabdominal ultrasound or CT angiography are equivocal.

Traditionally, serum and urine testing have played a small questionable role in diagnosing and prognosticating acute mesenteric ischemia; however, newer studies attempt to parse out which tests are helpful. There may be utility in preoperative lactate, creatinine, I-FAB, ischemia-modified albumin, procalcitonin, or IL-6 in determining the extent of disease and need for open surgery rather than endovascularization, but further studies need to be performed to corroborate this information. There may be some benefit to implementing abnormal lab values and imaging findings into diagnostic and prognostication scores, such as the CSI-score for chronic mesenteric ischemia, to determine which treatment modality would be preferable in different cases.

As the revascularization strategies of acute and chronic mesenteric ischemia continue to evolve and the popularity of endovascularization techniques increases, so does the importance of risk stratification and prognostication. Open surgery should remain the gold standard and preferred intervention for acute mesenteric ischemia for now, as follow-up laparotomy for necrosectomy is often required with endovascularization. There may be a role for hybrid interventions that involve staged approaches to occluded vessels, but more studies are needed to formulate standardized protocols. Adjunctive medical therapy for acute mesenteric ischemia may also be indicated as the temporizing benefits of preoperative prostaglandin infusions and albendazole have been appreciated in small human trials and animal models, respectively. More extensive trials in human patients are needed to determine their exact contribution and if preoperative infusions delay definitive revascularization.

In patients with chronic mesenteric ischemia, endovascular approaches are becoming the preferred treatment modality, and rightfully so. They are less invasive and have demonstrated comparable perioperative outcomes to open surgery. However, more research needs to be performed to determine patients who are at risk for in-stent stenosis and the need for reintervention, given their high late failure rate. These patients may benefit from open surgery as opposed to endovascular approaches. More studies are also needed to compare the outcomes of different open surgical techniques directly, as none have been recently performed, likely due to the popularity of endovascular approaches.

The high morbidity and mortality rates of both acute and chronic mesenteric ischemia urge clinicians to stay up to date with current optimal diagnostic and treatment strategies. Early recognition and intervention are vital to achieve ideal outcomes. The advent of new diagnostic and revascularization techniques for both acute and chronic conditions invites provides ample opportunity for future novel or supporting research. As illustrated above, more studies have been performed on acute mesenteric ischemia compared to chronic mesenteric ischemia, which invites future studies to focus on the latter. Large, prospective studies are needed to support the current smaller, retrospective studies given their inherent bias; however, understanding this may be difficult due to the rarity of the conditions.

## Data Availability

No new data was created or analyzed in this study.
